# Understanding Adult Participant and Parent Empowerment Prior to Evaluation in the Undiagnosed Diseases Network

**DOI:** 10.1007/s10897-018-0228-6

**Published:** 2018-03-01

**Authors:** Christina G. S. Palmer, Allyn McConkie-Rosell, Ingrid A. Holm, Kimberly LeBlanc, Janet S. Sinsheimer, Lauren C. Briere, Naghmeh Dorrani, Matthew R. Herzog, Sharyn Lincoln, Kelly Schoch, Rebecca C. Spillmann, Elly Brokamp, Undiagnosed Diseases Network

**Affiliations:** 1 Department of Psychiatry and Biobehavioral Sciences, UCLA Semel Institute, 760 Westwood Plaza, Room 47-422, Los Angeles, CA 90095 USA; 2 Department of Human Genetics, UCLA, Los Angeles, CA USA; 3 Institute for Society and Genetics, UCLA, Los Angeles, CA USA; 4 Department of Pediatrics, Duke University School of Medicine, Durham, NC USA; 5 Division of Genetics and Genomics, Boston Children’s Hospital, Boston, MA USA; 6 The Manton Center for Orphan Disease Research, Boston Children’s Hospital, Boston, MA USA; 7 Department of Pediatrics, Harvard Medical School, Boston, MA USA; 8 Department of Biomedical Informatics, Harvard Medical School, Boston, MA USA; 9 Department of Biostatistics, UCLA, Los Angeles, CA USA; 10 Department of Biomathematics, UCLA, Los Angeles, CA USA; 11 Department of Medical Genetics, Massachusetts General Hospital for Children, Boston, MA USA; 12 Department of Pediatrics, UCLA, Los Angeles, CA USA; 13 Division of Medical Genetics and Genomic Medicine, Vanderbilt University, Nashville, TN USA

**Keywords:** Empowerment, Support groups, Undiagnosed disease, Undiagnosed condition, Genetic counseling

## Abstract

The burden of living with an undiagnosed condition is high and includes physical and emotional suffering, frustrations, and uncertainty. For patients and families experiencing these stressors, higher levels of empowerment may be associated with better outcomes. Thus, it is important to understand the experiences of patients with undiagnosed conditions and their families affected by undiagnosed conditions in order to identify strategies for fostering empowerment. In this study, we used the Genetic Counseling Outcome Scale (GCOS-24) to assess levels of empowerment and support group participation in 35 adult participants and 67 parents of child participants in the Undiagnosed Diseases Network (UDN) prior to their UDN in-person evaluation. Our results revealed significantly lower empowerment scores on the GCOS-24 in adult participants compared to parents of child participants [*t*(100) = − 3.01, *p* = 0.003, average difference = − 11.12, 95% CI (− 3.78, − 18.46)] and no significant association between support group participation and empowerment scores. The majority of participants (84.3%, 86/102) are not currently participating in any support groups, and participation rates were not significantly different for adult participants and parents of child participants (11.4 vs. 19.7%, respectively, FE *p* = 0.40). Open-ended responses provided additional insight into support group participation, the challenges of living with undiagnosed conditions, and positive coping strategies. Future research will evaluate the extent to which empowerment scores change as participation in the UDN unfolds.

## Introduction

Rare diseases, when considered as a group, affect 25–30 million individuals in the US population (National Institutes of Health 2017), and more than half of those with rare diseases are children (EURORDIS 2018). Although roughly 80% of undiagnosed conditions are believed to be genetic in origin (Chong et al. 2015), for many patients and families, an underlying genetic cause is not found. As a result, too often, patients and families find themselves continuing on the diagnostic odyssey searching for answers (Molster et al. 2016; Zurynski et al. 2017).

The Undiagnosed Diseases Network (UDN; https://undiagnosed.hms.harvard.edu/) is a clinical research endeavor with the goal of ending the diagnostic odyssey for patients and families by identifying the underlying etiology of undiagnosed conditions. Participation in the UDN involves extensive phenotyping during a 1–5-day in-person evaluation at a participating UDN clinical site by a multidisciplinary team including clinical geneticists and genetic counselors during which biospecimen samples are taken and clinical tests are conducted; next-generation sequencing for most participants; and follow-up research, such as functional gene variant analyses (Gahl et al. 2015; Ramoni et al. 2017). The UDN is composed of seven clinical sites (Baylor College of Medicine, Duke University Health System with Columbia University Medical Center [Duke], Harvard Affiliated Hospitals (Brigham and Women’s Hospital, Massachusetts General Hospital, and Boston Children’s Hospital) [Harvard], Stanford Medical Center, University of California Los Angeles [UCLA], Vanderbilt University Medical Center [Vanderbilt], National Institutes of Health Undiagnosed Disease Program), two DNA sequencing cores (Baylor College of Medicine for whole exome sequencing, HudsonAlpha with Illumina for whole genome sequencing), a metabolomics core (Oregon Health and Science University and Pacific Northwest National Laboratory), a model organisms screening center (Baylor College of Medicine and University of Oregon), a biorepository (Vanderbilt University Medical Center), and a coordinating center (Harvard Medical School). In the UDN context, participants receive genetic counseling during the informed consent process to discuss the genetic testing options and possible results and during the in-person evaluation to discuss the available findings. All participants have the opportunity to ask questions and discuss clinical genetic testing that was done prior to their admission to the UDN. Some participants receive genetic counseling in the context of a diagnosis during the initial in-person evaluation, while for others, a diagnosis and updated genetic counseling may not occur until months or years after the in-person evaluation. Additional genetic counseling may occur after the end of the in-person evaluation based on the need of the participant and their families. The UDN provides an ideal venue to study the experience of living with an undiagnosed condition.

A recent study of applicants to the UDN provides some initial insight into the experiences of these patients and families (Spillmann et al. 2017). As part of the application process at the Duke clinical site, adult patients and parents of patients incapable of providing consent, including parents of children, were asked to include a written narrative describing their or their child’s medical history from their perspective. Analysis of 20 adult patient and 20 parent narratives found that consistent with other studies (Lewis et al. 2010; Madeo et al. 2012; Yanes et al. 2017; Zurynski et al. 2017), that the burden of living with an undiagnosed condition is high and includes physical and emotional suffering, frustrations, and much uncertainty.

Despite the commonalities between the narratives of adult patients and those of parents, there were also notable differences. This is a significant finding because, while there is some literature on parents’ experiences with undiagnosed conditions, there is a dearth of information on the experiences of adults with undiagnosed conditions. Spillmann and colleagues found that while both adult patients and parents were hopeful that the UDN experience would lead to a diagnosis, their thoughts about the impact of having a diagnosis differed. For the majority of adult patients, having a diagnosis meant being able to explore treatment options so that they could resume the lives they had led prior to onset of illness. For parents, however, having a diagnosis meant reducing uncertainty about what the future may hold and gaining information to improve management and quality of life for their children, consistent with previous research (Lewis et al. 2010; Zurynski et al. 2017). Both groups also noted frustration with medical providers, but for different reasons. For adult patients, this frustration often stemmed from the need to validate symptoms in light of nondiagnostic testing, while for parents it was caused by a concern that evidence crucial to making a diagnosis was being overlooked. Both parents and adult patients felt that they had exhausted all other diagnostic options. Similar to Lewis et al. (2010), parents also described the complexity of care required by their child and their role as an advocate to improve their child’s quality of life, even in the absence of a cure. While some parents expressed helplessness and distress at being unable to improve their child’s outcome, the majority emphasized their child’s positive attributes. Focusing “on the positive” has been observed in other studies of parents with a child with an undiagnosed condition as well (Lewis et al. 2010).

These narratives revealed how valuable and, in some cases, emotional it was for parents and adult patients to “tell their story,” suggesting that patients and families with undiagnosed conditions lack a support system or network of “similar others.” The findings that adult patients and parents experience undiagnosed conditions differently demonstrates the need to further understand and address the psychological impact of undiagnosed conditions in these groups in order to provide optimal support.

Patient empowerment is a key strategy to provide patients and parents with support. Empowerment is a process involving acquisition of knowledge and skills, utilization of resources, and involvement with similar others with the goal of enhancing outcomes and developing positive coping strategies (McConkie-Rosell and Sullivan 1999). Importantly, these are components in which medical providers can have a direct hand. Empirical data suggest that empowerment can improve cost-effective use of health services (Wallerstein 2006) and may lead to more informed decisions and positive outcomes for patients and families (McAllister et al. 2008).

Patient empowerment, as a process and as an outcome, has been studied in patients with chronic conditions (Bolen et al. 2014; Chen et al. 2013; Schmidt et al. 2015) and, more recently, in patients seeking genetic services (Diness et al. 2017; Inglis et al. 2015; McAllister 2016; McAllister et al. 2011). Since the majority of genetic conditions do not yet have treatment, studying patient empowerment in this cohort can provide insight into how the delivery of care itself influences empowerment.

There is some evidence that obtaining clinical genetics services increases patients’ sense of empowerment. Patient empowerment 2–4 weeks after an evaluation in a genetics clinic in the UK was higher than prior to the evaluation in a sample of patients with a variety of conditions, including those seeking a diagnosis (McAllister et al. 2011). In this study, levels of empowerment were not associated with type of condition, age, gender, ethnicity, or reason for referral. However, support group participation was associated with greater empowerment, highlighting the importance of providing this resource. In another study, increased empowerment 1-month post-psychiatric genetic counseling, a setting in which providers do not typically provide etiologic diagnoses, was also observed (Inglis et al. 2015). The results of these studies that include individuals with conditions that remained undiagnosed support the fact that there are components to the process of delivering care, distinct from identifying an underlying etiology, that are important for enhancing patient empowerment. Moreover, Inglis et al. (2015) found that individuals with and without a personal history of mental illness experienced increased empowerment, supporting the fact that there are benefits of the counseling process for individuals with and without disease.

These studies suggest that factors that contribute positively, or negatively, to patient and parent empowerment can be identified. Given the differences observed between adult patients and parents by Spillmann et al. (2017), evaluating these two groups individually may provide insights to guide interventions specific to each group. The UDN offers a unique opportunity to follow participants and parents going through a comprehensive clinical and research evaluation and to assess how, and to what extent, participants and parents become empowered by their experiences in the Network. In order to understand how the in-person evaluation may impact empowerment, it is critical to examine and understand the experience of this population at the beginning of their participation and to gain insight into baseline levels of empowerment, which can then be compared to empowerment levels after the evaluation. Thus, the purpose of our study was twofold: (1) to provide a description of UDN participant and parent levels of empowerment and support group participation prior to the UDN in-person evaluation and (2) to identify covariates of baseline empowerment in our UDN sample.

## Methods

### Study Design

The larger UDN empowerment study is a prospective longitudinal study of adult participant and parent empowerment with three assessment time points. The first assessment (T1) occurs after enrollment into the UDN and prior to the in-person evaluation; the second (T2) and third (T3) assessments occur 1 month and 1 year, respectively, after completion of the in-person evaluation. For this study of empowerment at baseline, we focus on T1 surveys completed between August 2016 and March 2017. The central Institutional Review Board (IRB) for the UDN, which is at the National Human Genome Research Institute (NHGRI), approved the study protocol.

### Participants

The study took place at four UDN clinical sites: Duke, Harvard, UCLA, and Vanderbilt. Adult UDN participants capable of providing consent and parents of UDN participants incapable of providing consent (i.e., minors, adults without capacity to consent) were invited to participate in the study. Non-English-using participants and biologically unrelated caregivers were excluded.

### Measures

The Genetic Counseling Outcome Scale (GCOS-24) was used to measure empowerment (McAllister et al. 2011). This patient-reported outcomes measure designed for clinical genetics services defines empowerment as the belief that one: (1) can make important life decisions in an informed way (decisional control); (2) has sufficient information about the condition, including risks to oneself and one’s relatives, and any treatment, prevention, and support available (cognitive control); (3) can make effective use of the health and social care systems for the benefit of the whole family (behavioral control); (4) can manage one’s feelings about having a genetic condition in the family (emotional regulation); and (5) can look to the future having hope for a fulfilling family life, for oneself, one’s family, and/or one’s future descendants (hope). The GCOS-24 has been validated in a clinical genetics population (*n* = 395) that included undiagnosed patients; has excellent internal consistency (Cronbach’s alpha = 0.87), test-retest reliability (*r* = 0.86), and convergent and divergent validities; and shows sensitivity to change over time with a medium-to-large effect size (McAllister et al. 2011). This measure has also been used to assess empowerment as an outcome of psychiatric genetic counseling (Inglis et al. 2015). Each of the 24 items in the measure is rated on a 7-point Likert scale ranging from 1 (strongly disagree) to 7 (strongly agree) with possible total scores ranging from 24 to 168. Higher scores indicate higher levels of empowerment.

We conducted a pilot study of this empowerment measure with 21 UDN participants, which demonstrated similar performance metrics as those described by McAllister et al. (2011). Based on pilot study feedback, the instructions were modified slightly to include the following: “Clinical genetics service refers to the UDN clinical visit. Condition refers to the reason you or your child is being evaluated in the UDN.”

For the final survey, an additional question was asked in multiple choice format to ascertain participation in support groups, and those who reported participation were asked to describe the group(s) in a free-response format. Participants were also encouraged to provide comments at the end of the survey. Demographic and family history information, including primary symptom category, was obtained from the data collected for the broader UDN protocol.

### Procedures

After enrollment and prior to the start of the UDN in-person evaluation, participants received an invitation email that contained a link to the T1 survey. When necessary, a paper and pencil version of the survey was made available in-person or by mail. During the consenting process for enrollment in the UDN, participants were informed about completing surveys; however, survey completion was voluntary and did not affect UDN participation.

## Data Analysis

As described by McAllister et al. (2011), relevant GCOS-24 items were reverse coded, and an overall empowerment score was calculated as the sum of the 24-item scores. Per the recommended procedure, missing data were filled in using simple imputation for surveys with greater than 75% completion (Inglis et al. 2015; McAllister et al. 2011) in order to compute a total empowerment score.

Pearson’s correlation and Fisher’s exact test were performed to evaluate association between quantitative or categorical variables, respectively. *T* tests were performed to compare group differences on quantitative variables. To explore participant group differences on individual GCOS-24 items, responses to each item were collapsed into three categories [disagree (containing strongly disagree, disagree, and slightly disagree responses), neutral, and agree (containing strongly agree, agree, slightly agree responses)] and assessed using Fisher’s exact test; imputed data were excluded from these analyses. Statistical significance was set at *p* < 0.05. No correction for multiple testing was used.

Free responses of the comments section were systematically analyzed using conventional content analysis with Atlas Ti (version 7; http://atlasti.com/). Content analysis allows for coding and categorization of the emerging themes without the use of theory (Hsieh and Shannon 2005; Satu et al. 2014). Initial coding was done by AMR. Once coding was complete, data were organized into the emerging themes and then reviewed by AMR and CP. Respondents were specifically asked if they belonged to support group(s) and, if so, to name it. These responses were then classified based on the type of support group.

## Results

### Demographic Characteristics

A total of 183 T1 surveys were distributed and 71% (130/183) were completed by participants prior to their UDN in-person evaluation from August 15th, 2016, to March 31st, 2017 (Duke: *n* = 34, Harvard: *n* = 31, UCLA: *n* = 31, Vanderbilt: *n* = 34). Among the remaining 53 participants who did not complete the survey, 12 were considered nonresponders because their in-person evaluations occurred before the analysis cutoff date, and 41 were considered to have pending surveys because their in-person evaluation was scheduled to occur after the analysis cutoff date.

Of the 130 surveys received, 27% (35/130) were completed by adult participants, 28% (36/130) were completed by mothers of participants, and 4% (5/130) were completed by fathers of participants. The remaining 54 surveys were completed by 27 mother-father dyads. Less than 1% of the GCOS-24 items were missing responses (25 out of 3120 item responses) with no single respondent missing more than 5 items. For those surveys with item responses missing (11%, 14/130), data were imputed. Internal consistency of the GCOS-24 items was assessed on 116 surveys with complete data, and the resulting Cronbach’s alpha of 0.84 was consistent with other studies using this measure (Inglis et al. 2015; McAllister et al. 2011).

In the 27 cases in which both parents filled out surveys, we found that the mother-father dyad empowerment scores were significantly correlated (*r* = 0.58, 95% CI 0.25, 0.78). Thus, in order to satisfy the assumption of independent observations underlying the *t* test, one parent was randomly excluded from each of the dyads for subsequent analysis. There also was one instance where both an adult female participant and her husband completed a survey. In this case, we retained only the adult participant’s survey for analysis to eliminate potential response dependency. This resulted in a final sample size of 102 respondents with a fairly even distribution across the four clinical sites (Duke: *n* = 25, Harvard: *n* = 26, UCLA: *n* = 23, Vanderbilt: *n* = 28).

Demographic characteristics of participants with an undiagnosed condition (adults and those incapable of providing consent) are presented in Table 1. The 35 adult participants are composed of 22 females and 13 males (mean age = 43.2 years, SD 15.8), and the 67 participants incapable of providing consent are composed of 32 females and 35 males (mean age = 9.3 years, SD 7.5). The primary race of the UDN participant was reported as Caucasian (88.2%, 90/102), with 96.1% (98/102) of UDN adult and child participants identifying as non-Hispanic. The predominant symptom category of the UDN participants was neurology (48.0%, 49/102).

**Table 1:** Demographic characteristics

	Total (*n* = 102)	Adult participants (*n* = 35)	Participants incapable of providing consent (*n* = 67)
Sex			
Females, *n* (%)	54 (52.9)	22 (62.9)	32 (47.8)
Males, *n* (%)	49 (47.1)	13 (37.1)	35 (52.9)
Age^a^	20.9 ± 19.5	43.2 ± 15.8	9.3 ± 7.5
Race			
Caucasian, *n* (%)	90 (88.2)	32 (94.1)	58 (86.6)
Black or African-American, *n* (%)	3 (2.97)	0 (0)	3 (4.5)
Asian, *n* (%)	3 (2.97)	0 (0)	3 (4.5)
Other, *n* (%)	5 (4.95)	2 (5.9)	3 (4.5)
Ethnicity			
Hispanic, *n* (%)	4 (3.9)	0 (0)	4 (6.5)
Non-Hispanic, *n* (%)	98 (96.1)	35 (100)	64 (94.1)
Primary symptom category			
Neurology, *n* (%)	49 (48.0)	11 (31.4)	38 (56.7)
Other, *n* (%)	11 (10.8)	4 (11.4)	7 (10.5)
Rheumatology, *n* (%)	7 (6.9)	4 (11.4)	3 (4.5)
Allergies, *n* (%)	6 (6.0)	4 (11.4)	2 (3.0)
Cardiology, *n* (%)	6 (6.0)	4 (11.4)	2 (3.0)
Musculoskeletal, *n* (%)	6 (6.0)	2 (5.7)	4 (6.0)
Gastroenterology, *n* (%)	5 (4.9)	0 (0)	5 (7.5)
N/A, *n* (%)	4 (3.9)	3 (8.6)	1 (1.5)
Endocrinology, *n* (%)	3 (2.9)	0 (0)	2 (3.0)
Ophthalmology, *n* (%)	1 (0.98)	1 (2.9)	0 (0)
Pulmonology, *n* (%)	1 (0.98)	1 (2.9)	(0)
Hematology, *n* (%)	1 (0.98)	0 (0)	1 (1.5)
Psychiatry, *n* (%)	1 (0.98)	0 (0)	1 (1.5)
Nephrology, *n* (%)	1 (0.98)	0 (0)	1 (1.5)
Support group participation, *n* (%)	N/A	4 (11.4)	N/A
Parental sex			
Females, *n* (%)	N/A	N/A	53 (77.1)
Males, *n* (%)	N/A	N/A	14 (20.8)
Parental age^a^	N/A	N/A	41.2 ± 8.5
Parental affected status, *n* (%)	N/A	N/A	3 (4.5)
Parental support group participation, *n* (%)	N/A	N/A	12 (19.7)

^a^The mean age ± standard deviation (in years) is indicated

Demographic characteristics of the parents of children with an undiagnosed condition are also presented in Table 1. For parent respondents, the mean age was 41.2 years (SD 8.5), and 4.5% (3/67) reported being affected. Five parents of adult participants incapable of consent completed the surveys and these responses are included in the parent sample.

For adult participant respondents, 42% (15/30) reported having children, 42% (15/30) reported not having children, and no information was available for the remaining five adult participants. Adult participants with children were significantly older (mean age = 47.5 years, SD 14.5) than those without children (mean age = 36.6 years, SD 12.4) [*t*(28) = − 2.23, *p* = 0.03]. Adult participants were less likely to fall into the “neurology” symptom category than children/participants incapable of providing consent (FE *p* = 0.02).

### Empowerment

The average score on the GCOS-24 measure was 112.66 (SD 18.42, min 69, max 156). Scores were significantly lower for adult participants (mean = 105.4, SD 16.94) than for parents (mean = 116.5, SD 18.1) [*t*(100) = − 3.01, *p* = 0.003, average difference = − 11.12, 95% CI (− 3.78, − 18.46)]. To further evaluate this apparent distinction between adult participants and parents, we compared the baseline GCOS-24 scores from our respondents to scores obtained from adults with a psychiatric illness of unknown etiology prior to genetic counseling (*n* = 45, mean = 109.20, SD 14.7) (Inglis et al. 2015). There was no significant difference between the UDN adult participants and adults with a psychiatric illness [*t*(67) = 1.08, *p* = 0.28, average difference = 3.89, 95% CI (− 3.31, 11.09)]; however, the adults with a psychiatric illness had significantly lower scores than our UDN parents [*t*(105) = − 2.32, *p* = 0.02, average difference = − 7.21, 95% CI (− 13.38, − 1.04)].

Because five items ask specifically about (future) children, we assessed if the score difference was an artifact produced by responses of adult participants without children. To evaluate this, responses from these five items were excluded and new scores were calculated for all respondents. Scores remained significantly lower for adult participants (mean = 81.6, SD 14.65) than for parents (mean = 90.87, SD 15.4) [*t*(100) = − 2.93, *p* = 0.004; average difference = − 9.27, 95% CI (− 3.0, − 15.54)].

Empowerment scores were not associated with respondent gender [*t*(98) = 0.93, *p* = 0.35], support group participation [*t*(94) = − 0.11, *p* = 0.91], age [*r* = − 0.04, *p* = 0.72], or the primary symptom category of the UDN participant [*t*(100) = 0.71, *p* = 0.48]. In addition, in a separate analysis of parents, their scores were not associated with parental status (mother/father) [*t*(66) = 0.15, *p* = 0.88], their child’s age [*r* = − 0.07, *p* = 0.58], child’s gender [*t*(65) = − 1.37, *p* = 0.18], or child’s race (Caucasian/other) [*t*(65) = 0.85, *p* = 0.40].

The response distribution for each item on the GCOS-24 was compared between adult participants and parents to gain further insight into group differences. Table 2 lists the GCOS-24 items in descending order of percent agreement for the adult participants and identifies the items for which there are significant differences in response distribution between the two groups. Examination of specific items on the empowerment measure revealed that nearly all participants and parents understood why they were referred to the UDN for evaluation. In addition, the majority of respondents felt that they could explain what the condition meant to their family and others, could cope with having the condition in the family, felt positive about the future, could make plans and decisions, and understand the impact of the condition on (future) children. However, more than half of respondents also felt that the condition was upsetting and that they experience uncertainty about other family members having the condition.

**Table 2:** GCOS-24 item distributions for UDN adult participants and parents of UDN participants unable to provide consent

Item	Adult participants (*n* = 35)			Parents (*n* = 67)			*p* ^a^
	Agree % (*n*)	Neutral % (*n*)	Disagree % (*n*)	Agree % (*n*)	Neutral % (*n*)	Disagree % (*n*)	
[1^b^] I am clear in my own mind why I am attending the clinical genetics service.	97.1 (34)	0	2.9 (1)	98.5 (66)	0	1.5 (1)	1.0
[14] I understand the reasons why my doctor referred me to the clinical genetics service.	97.1 (34)	2.9 (1)	0	97.0 (65)	1.5 (1)	1.5 (1)	1.0
[23] I understand what concerns brought me to the clinical genetics service.	91.4 (32)	5.7 (2)	2.9 (1)	95.5 (64)	1.5 (1)	3.0 (2)	0.55
[2] I can explain what the condition means to people in my family who may need to know.	85.7 (30)	0	14.3 (5)	82.1 (55)	6.0 (4)	11.9 (8)	0.44
[9] I am able to cope with having this condition in my family.	70.6 (24)	11.8 (4)	17.7 (6)	78.8 (52)	7.6 (5)	13.6 (9)	0.58
[18] I don’t know who else in my family might be at risk for this condition (reverse coded item).	69.7 (23)	18.2 (6)	12.1 (4)	60.6 (40)	15.2 (10)	24.2 (16)	0.38
[16] I can explain what the condition means to people outside my family who may need to know (e.g., teachers, social workers).	68.6 (24)	11.4 (4)	20.0 (7)	77.6 (52)	9.0 (6)	13.4 (9)	0.57
[11^c^] Having this condition in my family makes me feel anxious.	62.9 (22)	14.3 (5)	22.9 (8)	61.2 (41)	14.9 (10)	23.9 (16)	1.0
[8] I feel positive about the future	60.0 (21)	20.0 (7)	20.0 (7)	61.2 (41)	17.9 (12)	20.9 (14)	0.96
[17^c^] I don’t know what I can do to change how this condition affects me/my children.	60.0 (21)	25.7 (9)	14.3 (5)	46.3 (31)	22.4 (15)	31.3 (21)	0.17
[20] I am able to make plans for the future.	58.8 (20)	11.8 (4)	29.4 (10)	70.2 (47)	9.0 (6)	20.9 (14)	0.46
[3] I understand the impact of the condition on my child(ren)/any child I may have.	57.1 (20)	28.6 (10)	14.3 (5)	71.6 (48)	13.4 (9)	14.9 (10)	0.19
[24] I can make decisions about my condition that may change my child(ren)’s future/the future of any child(ren) I may have.	57.1 (20)	31.4 (11)	11.4 (4)	70.8 (46)	18.5 (12)	10.8 (7)	0.29
[12^c^] I don’t know if this condition could affect my other relatives (brothers, sisters, aunts, uncles, cousins).	55.9 (19)	8.8 (3)	35.3 (12)	48.5 (32)	13.6 (9)	37.9 (25)	0.69
*[19] I am hopeful that my children can look forward to a rewarding family life.*	*54.3 (19)*	*45.7 (16)*	*0*	*83.6 (56)*	*10.5 (7)*	*6.0 (4)*	*0.0001*
*[15] I know how to get the non-medical help I/my family needs (*e.g., *educational, financial, social support).*	*51.4 (18)*	*11.4 (4)*	*37.1 (13)*	*73.1 (49)*	*10.5 (7)*	*16.4 (11)*	*0.047*
[4^c^] When I think about the condition in my family, I get upset.	50.0 (17)	20.6 (7)	29.5 (10)	52.2 (35)	22.4 (15)	25.4 (17)	0.92
[5^c^] I don’t know where to go to get the medical help I/my family need(s).	48.6 (17)	2.9 (1)	48.6 (17)	31.3 (21)	13.4 (9)	55.2 (37)	0.11
[22^c^] I am powerless to do anything about this condition in my family.	42.9 (15)	20.0 (7)	37.1 (13)	40.3 (27)	14.9 (10)	44.8 (30)	0.70
*[10* ^*c*^ *] I don’t know what could be gained from each of the options available to me.*	*36.4 (12)*	*36.4 (12)*	*27.3 (9)*	*15.4 (10)*	*32.3 (21)*	*52.3 (34)*	*0.026*
*[21* ^*c*^ *] I feel guilty because I (might have) passed this condition on to my children.*	*36.4 (12)*	*45.5 (15)*	*18.2 (6)*	*34.3 (23)*	*17.9 (12)*	*47.8 (32)*	*0.003*
[13^c^] In relation to the condition in my family, nothing I decide will change the future for my children/any children I might have.	14.3 (5)	40.0 (14)	45.7 (16)	24.2 (16)	21.2 (14)	54.6 (36)	0.12
*[6] I can see that good things have come from having this condition in my family.*	*11.4 (4)*	*22.9 (8)*	*65.7 (23)*	*40.0 (26)*	*21.5 (14)*	*38.5 (25)*	*0.006*
[7] I can control how this condition affects my family.	8.6 (3)	14.3 (5)	77.1 (27)	25.8 (17)	18.2 (12)	56.1 (37)	0.07

^a^Fisher’s exact test, items with group differences at *p* ≤ 0.05 are italicized. Due to missing data, total number of responses per item can vary

^b^Numbering of items in the survey

^c^Reverse coded for computing overall empowerment score

Group differences were also observed. Compared to adult participants, parents were more likely to report agreement on “I am hopeful that my children can look forward to a rewarding family life” (*p* = 0.0001), “I know how to get the non-medical help I/my family needs (e.g. educational, financial, social support)” (*p* = 0.047), and “I can see that good things have come from having this condition in my family” (*p* = 0.006). Compared to adult participants, parents were more likely to disagree with “I don’t know what could be gained from each of the options available to me” (*p* = 0.026). Parents were less likely to report a neutral response and more likely to disagree with the statement “I feel guilty because I (might have) passed this condition on to my children” (*p* = 0.003).

### Support Group Participation

The majority of respondents indicated that they do not participate in any support groups (84.3%, 86/102). In addition, the rates of participation were not significantly different for adult participants and parents of child participants (11.4 vs. 19.7%, respectively, FE *p* = 0.40) (Table 1).

A total of 33 support groups were described by the 16 respondents (4 adult participants, 12 parents) who reported support group participation. Coding of these groups suggests six categories of support groups: general, special needs, symptom focus, specific disease, rare disease, and undiagnosed disease (Table 3). The categories with the largest number of support groups identified were special needs (24%, 8/33) and rare disease (24%, 8/33), followed by general (21.1%, 7/33), symptom focus (18.2%, 6/33), specific disease (6%, 2/33) and undiagnosed disease (6%, 2/33). Only two respondents, both parents, reported participating in support groups in the rare disease category. Parental participation is reflected in all six support group categories, while adult participation is reflected in four of the support group categories. It was not unusual for respondents to participate in more than one support group (100%, 4/4 adults; 33.3%, 4/12 parents) and more than one *category* of support group (75%, 3/4 adults; 33.3%, 4/12 parents). A higher percentage of adult participants were involved in “General Support” groups than parents (75 vs. 16.7%, respectively) and a higher percentage of parents, particularly mothers, were involved in the “special needs” groups than adult participants (50 vs. 0%, respectively). Only two respondents (12.5%) reported participation in groups designed for individuals impacted by undiagnosed conditions.

**Table 3:** Support group participation

Type of support	Specific examples	Respondents
General	The Cause (church) Telephone support groups Local support group Online forums Community support Parent support group Individual and marriage counseling	[Participant 89—adult] [Participant 45—adult] [Participant 45—adult] [Participant 92—adult] [Participant 92—adult] [Participant 46—mom] [Participant 83—mom]
Special needs	Facebook groups Friends of children with complex conditions Educators and therapists at school for complex children Special Education Parents Advisory Council Local Special Olympics chapter Online support group Jewish mothers of children with special needs Physician Mothers of Children with Special Needs	[Participant 62—mom] [Participant 10—mom] [Participant 10—mom] [Participant 22—mom] [Participant 22—mom] [Participant 66—mom] [Participant 104—mom] [Participant 18—mom]
Symptom focus	Friends of Brain Injury Spinal cord disease online support group Auto-inflammatory Alliance Facebook page Online pediatric epilepsy support groups Infantile spasms group Facebook group-hypotonia parents connection	[Participant 89—adult] [Participant 45—adult] [Participant 121—mom] [Participant 22—mom] [Participant 18—mom] [Participant 96—dad]
Specific disease	National Urea Cycle Disorders Foundation Marinesco-Sjogren Syndrome Support Group	[Participant 10—mom] [Participant 64—dad]
Rare disease	Utah Rare Wisconsin Rare RunmyDNA.com NORD Every Life Foundation for Rare Diseases Rare Disease Legislative Advocates Global Genes Rare disease community for connection and support	[Participant 49—adult] [Participant 83—mom]
Undiagnosed disease	Rare and Undiagnosed Disease Network San Diego Undiagnosed Family Support Group	[Participant 49—adult] [Participant 87—dad]

### Participant Comments

Twenty-seven participants (26.5%), comprised of 11 adult participants (31.4%, 11/35) and 16 parents (23.9%, 16/67), provided comments at the end of the survey. Adult participants were not significantly more likely to provide responses than the parents (FE *p* = 0.48). Responses were categorized into five themes, with representation from adult participants and parents in all themes. Two themes involved the UDN itself: “Reasons for applying to the UDN” and “UDN program/process.” These comments generally reflected hope for the end of the diagnostic odyssey and appreciation for a program dedicated to individuals with undiagnosed conditions. Two themes captured emotional states brought on by living with an undiagnosed condition, such as feelings of uncertainty, and evidence of positive coping. These themes were labeled “challenges living undiagnosed” and “positive coping strategy.” A fifth theme, labeled “explaining survey responses,” reflected participants’ comments about why they did or did not respond to a survey item in a certain way. Table 4 provides illustrative comments for each category.

**Table 4:** Themes from open-ended survey responses

Theme	Adult participants	Parents
Reasons for applying to the UDN	“…hope that even if I don’t find answers for my own condition, that I might be able to help someone else.” [participant 3—adult] “For me an unsolved problem is an exciting opportunities for scientific advancement.” [participant 24—adult] “This testing for me is my last go to try to figure out what I really have. I’ve been disappointed before with testing and this is just another opportunity to find out more or nothing at all. I just want to know why I’m the only one who has this problem and if I have a child will they get the bad gene that I have.” [participant 44—adult]	“As a mother my reasoning for wanting [my son] to be part of the study is to get some answers as to the why his medical problems developed. I understand that a “cure” is not the objective. If his medical problems can be given a name, a link, and a possible understanding of the progression and what could potentially go wrong would be vital information not just for [my son] but his sister and [his] future potential children. I as a mother just want [my son] to live a long happy life.” [participant 16—mom] “I hope one day he has a diagnosis. I hope one day we can help him so that we aren’t afraid of what happens when he gets sick. I hope that one day when someone asks me what happened to him, I can better explain why he is diifferent…” [participant 61—mom]
UDN program/process	“It is about 2 weeks before my clinic visit and I must say that everyone has been amazing and so helpful. I could not ask for a better experience thus far. I am thankful and so grateful for this opportunity…” [participant 3—adult] “I just wanted to thank all of the people who are in charge of this research study, your [sic] going to change a lot of lives for the better and this could be the one thing that could change my life if something comes out of the study.” [participant 44—adult]	“I am very thankful for UDN.” [participant 21—mom]
Challenges living undiagnosed	“We say that we live in a diagnostic odyssey times four. Being the mother of three children that suffer with an undiagnosed rare disease while being undiagnosed as well, is very difficult.” [participant 49—adult] “Our family is our support group, because no one else has this condition and no one else can even relate to this experience we have been through” [participant 111—adult]	“It creates a lot of anxiety because my daughter can’t communicate with me or the doctors. I feel responsible for [my daughter’s] outcomes because it seems like I’m always the one who knows when there is a problem… It is stressful to make plans because one never knows when something is going to go wrong, this has been worse since [my daughter] was diagnosed with epilepsy, then a tumor and then infection. I asked the doctor to put in the letter for [my daughter] because it just seemed like there was something more going on with her but no doctor is looking at the whole picture.” [participant 4—mom] “The hardest thing for me is not knowing what lies ahead. He continually exceeds all of my expectations! But, what happened? Why did he get sick? What if he gets sick again? How do I help him? Not having answers to these questions, makes me feel powerless.” [participant 61—mom] “I am worried that there could be no cure or treatment for my daughter’s medical condition, and that that will affect her learning, her normal life and on adulthood her independent living. I would like my daughter to one day be able to go to college and have a normal life. Worried of what would happen to her when us, as parents, no longer exist, if she makes no progress.” [participant 110—dad]
Positive coping strategy	“If nothing happens then I’ll just live my life the way I’ve been living which is unsure of what to do moving forward with my life but not to give up.” [participant 44—adult]	“As I have explained to [my son] throughout his young life some people have to do maintenance on their body more so than others to keep it running well, like a car. We cannot control if things go wrong, but we can do our best to try to prevent further problems. If that involves continuous doctor appointments, medications, procedures and surgeries than that is what we must do. It will not interfere with having a happy fulfilling life. As the study goes, [my son] understands knowledge is power, and whatever we can learn about his medical conditions can only give us power in making the best decisions possible for his medical care and future. Thank you” [participant 16—mom] “We are very fortunate to have met so many people that have advocated and continue to support [our daughter] and our family. We have many therapists and medical professionals and teachers that have witnessed [our daughter’s] episodes and want to help and we are so grateful for them.” [participant 47—mom] “I don’t have a good diagnosis for my son. However, I know my son. I know he is a fighter and has overcome alot. I know that he is surrounded by people that will help him.” [participant 61—mom] “I am so blessed to have him in my life! He is inspirational to everyone he meets!... He continually exceeds all of my expectations!... I find strength knowing that my son is a fighter. I know that nothing stands in his way! He is a child of God! He is a survivor! My son is a miracle!!” [participant 61—mom]
Explaining survey responses	“…recently, in fact on the same day I was accepted into UDN, I was terminated from a treatment program that managed some of my most debilitating and life threatening symptoms. While working hard to find a substitute treatment, a treatment alternative, am experiencing much higher levels of anxiety. If I completed the survey just 2 weeks ago, would provide different answers.” [participant 24—adult] “I do not have any children. Therefore, I answered “neither agree nor disagree” on all questions involving children.” [participant 45—adult]	“Since we do not know what the specific condition is, it is difficult to answer many of the questions since so much is unknown.” [participant 59—mom] “Thank you for asking these questions. It matters. I think about these issues daily.” [participant 66—mom]

## Discussion

The purpose of this study was to investigate UDN participant and parent empowerment prior to the UDN in-person evaluation in order to better understand baseline levels of empowerment in this population. Using the GCOS-24 measure (McAllister et al. 2011), we found that adults have significantly lower empowerment levels than parents of children with undiagnosed conditions. Although these groups differed in the proportion of participants affected with primarily neurological symptoms, there was no evidence that empowerment scores were explained by primary symptom category or other demographic variables, including respondent and child age and gender. Overall, these results are consistent with the findings of Spillmann et al. (2017) and provide further evidence that the experience of living with an undiagnosed condition differs between adult patients and parents of patients.

In our sample, the average GCOS-24 empowerment score was 112.67 (SD 18.33), with a parental average score of 116.5 (SD 18.1) and an adult average score of 105.4 (SD 16.94). In a study of 61 individuals with suspected genetic conditions (cancer, intellectual disability/cytogenetics, eye disorders, other) referred for a genetics consultation in Denmark, the average GCOS-24 empowerment score was 120.8 (SD 15.1) prior to consultation (Diness et al. 2017). In a study of individuals referred to one of five clinical genetics services in the UK (sample sizes ranging from 42 to 74), the average GCOS-24 empowerment scores ranged from 104 to 121 (SD 8.9–25.8) across the five centers (McAllister 2016). Finally, in a sample of 45 adults in Canada with a personal history of psychiatric illness for which the etiology is unknown, the average GCOS-24 score prior to genetic counseling was 109.29 (SD 14.7) (Inglis et al. 2015). Of note, we found that empowerment levels of the adults with a psychiatric illness are similar to levels of UDN adult participants and significantly lower than those of UDN parents, further bolstering the evidence that the experience of living with an undiagnosed condition differs between adult patients and parents. In light of our finding of the importance of distinguishing between adult patients and parents of a child, we did not compare GCOS-24 scores with the other two previous studies (Diness et al. 2017; McAllister 2016) because insufficient information about those patient samples limited our ability to interpret the results. However, overall, it does appear that the empowerment levels of our overall UDN cohort fall within the range reported in studies of patients/families with a variety of conditions referred for genetic evaluation or genetic counseling.

Responses to specific items on the empowerment measure revealed that nearly all participants and parents understood why they were referred to the UDN for evaluation. In addition, the majority of respondents felt that they could explain what the condition meant to their family and others, could cope with having the condition in the family, felt positive about the future, could make plans and decisions, and understand the impact of the condition on (future) children. The ability to cope, make plans, and feel positive about the future in our sample are consistent with a recent report of the psychosocial profile of parents of a child with an undiagnosed condition at the Duke UDN Clinical Site in which parents were found to have high coping self-efficacy, engagement in their child’s healthcare, and tolerance for uncertainty (McConkie-Rosell et al. 2018). Additional research in adults with an undiagnosed condition is needed to ascertain if they have a similar psychosocial profile.

More than half of our respondents also felt that the condition was upsetting and that they experience uncertainty about other family members having the condition. This finding is consistent with the suffering, frustration, and uncertainty described in the narratives of patients applying to the UDN (Spillmann et al. 2017) and the finding that more than a third of parents of a child with an undiagnosed condition participating at the Duke UDN Clinical Site meet criteria for mild to moderate depression or anxiety (McConkie-Rosell et al. 2018). The partial overlap of participants across these studies offers a more comprehensive picture of the challenges associated with undiagnosed conditions, particularly for parents. These results suggest possible sites of intervention to address these concerns.

Item responses also revealed differences between adult participants and parents. Not only were the majority of parents more hopeful for their child’s future compared to adult participants, parents were also significantly more likely to report knowing how to get nonmedical help and what can be gained from available options. These findings suggest several possibilities, including that more support may be available for parents of children with medical issues, that parents are more able to access support than adult participants because the adults do not feel well, or, extrapolating from the work of Spillmann et al. (2017), that adults with undiagnosed conditions are concerned about having to continue validating their illness if they try to reach out for additional help. Parents were also significantly more likely to report seeing good things come from having the condition in the family and feeling a sense of control over how the condition affects the family, aspects that may be associated with better coping and adaptation (Madeo et al. 2012). Overall, these differences suggest that, compared to adult participants’ levels of empowerment related to the potential genetic aspects and chronic issues of their own condition, parents enter the UDN process with increased levels of empowerment related to these aspects of their child’s condition across a variety of the domains, including hope, emotional regulation, and cognitive and behavioral control.

In our sample, 15.7% (16/102) of respondents reported support group involvement, with adult participants and parents (primarily mothers) equally likely to be involved. This rate is lower than the 53.5% (299/620) of adults with a rare disease diagnosis in Australia who reported using a support group in the last 12 months (Molster et al. 2016), and suggests that lack of a diagnosis may hinder support group participation. Also of note, in our sample, there was no evidence of an association between empowerment levels and support group participation, contrary to previous studies (Bartlett and Coulson 2011; McAllister et al. 2011).

The goals of support groups include helping people cope with their disease, facilitating social interactions and networking, and encouraging group advocacy (Finlayson and Cho 2011). At their essence, support groups provide an avenue for personal contact with other individuals who have gone through a similar experience. One possible explanation for the lack of association between support group participation and empowerment in our UDN sample is that groups available to patients and families with undiagnosed conditions cannot meet the need for a shared experience. A study of the impact of online support groups on empowerment processes and outcomes in individuals with chronic disease found that the most common empowerment process was associating and finding similarities with another support group member (Bartlett and Coulson 2011). The ability to find commonalities with other members was associated with a number of empowerment outcomes, including feeling better informed, more confident in the physician and treatment, more acceptance of illness, and more optimism and hope for the future. By virtue of being affected with an *undiagnosed* condition, patients and families may have difficulty identifying support groups that offer a similar shared experience. The ability to find commonalities often ends with the sharing of the search for a diagnosis and the experience of frustration of the diagnostic odyssey. As a result, empowerment processes may be inhibited. In our study, one respondent specifically commented on the challenge of finding a support group relevant to the needs of someone with an undiagnosed condition—a challenge that has been previously noted (Lewis et al. 2010).

Regardless of the challenges, for some UDN participants and family members, the desire to find individuals with similar experiences is strong, evident by reported support group involvement. These support groups fell into six categories: “general,” “special needs,” “symptom focus,” “specific disease,” “rare disease,” and “undiagnosed disease.” The categories with the greatest participation included “special needs” and “rare disease”, although only two participants listed participation in the “rare disease” category. While more adults participated in “general” support groups than parents and more parents participated in “special needs” support groups than adults, overall adult and parental participation was reflected in all or most of the support group categories. These results suggest that no single support group or support group category completely addresses the needs of individuals and families impacted by undiagnosed conditions. In fact, among the categories of support groups identified by our UDN participants and parents, support groups for undiagnosed conditions were the least frequently cited, suggesting a need for groups for individuals impacted by undiagnosed conditions. In order to address this and other needs of those affected by undiagnosed conditions, the UDN recently created a group of participants, called the Participant Engagement and Empowerment Resource (PEER). Future research is needed to evaluate the impact of PEER on empowerment levels of adult patients and parents participating in the UDN.

Assessment of open-ended comments at the end of the survey provided additional insight into our cohort. Comments in the two UDN-related themes generally reflected hope for the end of the diagnostic odyssey and appreciation for a program dedicated to individuals with undiagnosed conditions. Because investigation of rare and undiagnosed conditions requires multidisciplinary teams involving clinicians, genetic counselors, and basic researchers, the UDN-related themes highlight the importance, from the patient point of view, of developing programs and strategies that bridge the clinical research gap to advance patient care. Two other themes captured emotional states brought on by living with an undiagnosed condition, like feelings of uncertainty, and evidence of positive coping. Findings of hope and positive coping were also observed in the set of narratives written by UDN applicants or parents on behalf of a child (Spillmann et al. 2017) and as part of a psychosocial profile of parents of a child with an undiagnosed disorder (McConkie-Rosell et al. 2018).

### Limitations

Patient empowerment provides one useful strategy for conceptualizing and measuring outcomes of evaluation in an undiagnosed disease clinic setting. However, in the open-ended survey responses, some participants noted that it was challenging to respond to GCOS-24 items related to children for individuals without children and items related to “condition” for those of whom “condition” implies “diagnosis.” Furthermore, the GCOS-24 operationalization of patient empowerment may not fully capture all of the important aspects of living with an undiagnosed condition, or all of the outcomes from evaluation in an undiagnosed disease clinic setting. Future research could address if there are better measures of patient empowerment for this population. We, however, found that the GCOS-24 measure had excellent internal consistency, high completion rates, and scores that fall within a range of three other studies using this measure in populations that included individuals with potential genetic conditions, suggesting that the GCOS-24 performs reasonably well in a population impacted by undiagnosed conditions.

This study sample is composed of adults and parents who applied to the UDN, which takes time and effort. As a result, our findings may not generalize to all individuals and families impacted by undiagnosed conditions. In addition, because our participants completed the survey after consent involving considerable interaction with UDN staff, usually a genetic counselor, it is possible that their baseline empowerment scores are higher than if they had completed the survey prior to any interaction with the UDN team. As a result, effect sizes for changes in empowerment later in the UDN process may be weakened. We also did not correct for multiple testing when we examined each of the GCOS-24 items for participant group differences because it is difficult to know how best to do this when the items of a scale are not independent. Additional research with larger samples can provide further insight into the robustness of these group differences. Finally, the sample is composed primarily of Caucasian individuals, and so generalizability to other groups should be done with caution.

## Implications for Genetic Counseling

Our results indicate that adult patients and parents have different experiences living with undiagnosed conditions. This understanding can be applied when establishing rapport and identifying needs as part of genetic counseling practice, whether counseling patients with undiagnosed conditions within a regular clinical genetics service or within an undiagnosed disease clinic setting. This work also revealed that a small percentage of patients and parents affected by undiagnosed conditions participate in support groups and that, for the most part, these support groups do not specifically serve the undiagnosed. As genetic counseling involves providing resources for patients, genetic counselors can consider taking a proactive role in developing support groups or other methods for supporting patients and families affected by undiagnosed conditions.

## Future Research

Given the evidence of increased empowerment several weeks after genetic counseling and evaluation (Inglis et al. 2015; McAllister et al. 2011), we hypothesize that adult patients and parents will experience increased empowerment following the UDN in-person evaluation, which will need to be assessed. Future research on support group participation is also warranted. Given the small number of individuals reporting participation in support groups in this study, replication of this finding in a larger sample is essential. We did not ascertain whether or not participants wanted to be part of a support group. However, parents of a child with a rare disease have been repeatedly shown to identify access to a support group as an important means of support (Pelentsov et al. 2015). Hence, research is also needed to explore reasons why participants and parents of children with undiagnosed conditions participate or not in a support group. Since support group participation has been shown to increase levels of empowerment in other studies, examining the role of support groups or other resources specific to patients and families affected by undiagnosed conditions would be beneficial. In addition, the impact of different resources and strategies on adult patient and parent levels of empowerment should be measured.

## Conclusion

This study demonstrated that, prior to the UDN in-person evaluation, parents of children with undiagnosed conditions have higher levels of empowerment related to potential genetic and chronic aspects of a condition than adult participants. Our findings suggest a need for support group resources specifically designed for patients and families impacted by undiagnosed conditions, tailored differently for adult patients and parents. Future research is needed to determine if there are specific factors that influence empowerment during UDN participation and, if these factors are identified, developing targeted approaches in the UDN protocol to increase empowerment. Ideally, this work will provide a foundation for improving levels of empowerment, determining the extent to which patient/family empowerment influences downstream outcomes, and developing protocols for enhancing patient empowerment in clinics devoted to individuals with undiagnosed conditions nationally and worldwide.

## Consortia

Members of the Undiagnosed Diseases Network are David R. Adams, Mercedes E. Alejandro, Patrick Allard, Euan A. Ashley, Mahshid S. Azamian, Carlos A. Bacino, Ashok Balasubramanyam, Hayk Barseghyan, Gabriel F. Batzli, Alan H. Beggs, Hugo J. Bellen, Jonathan A. Bernstein, Anna Bican, David P. Bick, Camille L. Birch, Devon Bonner, Braden E. Boone, Bret L. Bostwick, Lauren C. Briere, Donna M. Brown, Matthew Brush, Elizabeth A. Burke, Lindsay C. Burrage, Shan Chen, Gary D. Clark, Terra R. Coakley, Joy D. Cogan, Cynthia M. Cooper, Heidi Cope, William J. Craigen, Precilla D'Souza, Mariska Davids, Jean M. Davidson, Jyoti G. Dayal, Esteban C. Dell'Angelica, Shweta U. Dhar, Ani Dillon, Katrina M. Dipple, Laurel A. Donnell-Fink, Naghmeh Dorrani, Daniel C. Dorset, Emilie D. Douine, David D. Draper, Annika M. Dries, David J. Eckstein, Lisa T. Emrick, Christine M. Eng, Gregory M. Enns, Ascia Eskin, Cecilia Esteves, Tyra Estwick, Liliana Fernandez, Paul G. Fisher, Brent L. Fogel, Noah D. Friedman, William A. Gahl, Emily Glanton, Rena A. Godfrey, David B. Goldstein, Sarah E. Gould, Jean-Philippe F. Gourdine, Catherine A. Groden, Andrea L. Gropman, Melissa Haendel, Rizwan Hamid, Neil A. Hanchard, Lori H. Handley, Matthew R. Herzog, Ingrid A. Holm, Jason Hom, Ellen M. Howerton, Yong Huang, Howard J. Jacob, Mahim Jain, Yong-hui Jiang, Jean M. Johnston, Angela L. Jones, David M. Koeller, Isaac S. Kohane, Jennefer N. Kohler, Donna M. Krasnewich, Elizabeth L. Krieg, Joel B. Krier, Jennifer E. Kyle, Seema R. Lalani, C. Christopher Lau, Jozef Lazar, Brendan H. Lee, Hane Lee, Shawn E. Levy, Richard A. Lewis, Sharyn A. Lincoln, Allen Lipson, Sandra K. Loo, Joseph Loscalzo, Richard L. Maas, Ellen F. Macnamara, Calum A. MacRae, Valerie V. Maduro, Marta M. Majcherska, May Christine V. Malicdan, Laura A. Mamounas, Teri A. Manolio, Thomas C. Markello, Ronit Marom, Julian A. Martínez-Agosto, Shruti Marwaha, Thomas May, Allyn McConkie-Rosell, Colleen E. McCormack, Alexa T. McCray, Jason D. Merker, Thomas O. Metz, Matthew Might, Paolo M. Moretti, John J. Mulvihill, Jennifer L. Murphy, Donna M. Muzny, Michele E. Nehrebecky, Stan F. Nelson, J. Scott Newberry, John H. Newman, Sarah K. Nicholas, Donna Novacic, Jordan S. Orange, J. Carl Pallais, Christina GS. Palmer, Jeanette C. Papp, Neil H. Parker, Loren DM. Pena, John A. Phillips III, Jennifer E. Posey, John H. Postlethwait, Lorraine Potocki, Barbara N. Pusey, Chloe M. Reuter, Amy K. Robertson, Lance H. Rodan, Jill A. Rosenfeld, Jacinda B. Sampson, Susan L. Samson, Kelly Schoch, Molly C. Schroeder, Daryl A. Scott, Prashant Sharma, Vandana Shashi, Edwin K. Silverman, Janet S. Sinsheimer, Kevin S. Smith, Ariane G. Soldatos, Rebecca C. Spillmann, Kimberly Splinter, Joan M. Stoler, Nicholas Stong, Jennifer A. Sullivan, David A. Sweetser, Cynthia J. Tifft, Camilo Toro, Alyssa A. Tran, Tiina K. Urv, Zaheer M. Valivullah, Eric Vilain, Tiphanie P. Vogel, Daryl M. Waggott, Colleen E. Wahl, Nicole M. Walley, Chris A. Walsh, Michael F. Wangler, Patricia A. Ward, Katrina M. Waters, Bobbie-Jo M. Webb-Robertson, Monte Westerfield, Matthew T. Wheeler, Anastasia L. Wise, Lynne A. Wolfe, Elizabeth A. Worthey, Shinya Yamamoto, Yaping Yang, Guoyun Yu, Diane B. Zastrow, Chunli Zhao, Allison Zheng
